# Growth Mechanism Studies of ZnO Nanowires: Experimental Observations and Short-Circuit Diffusion Analysis

**DOI:** 10.3390/nano7070188

**Published:** 2017-07-21

**Authors:** Po-Hsun Shih, Sheng Yun Wu

**Affiliations:** Department of Physics, National Dong Hwa University, Hualien 97401, Taiwan; libra.kevin.t@gmail.com

**Keywords:** nanocrystalline materials, zinc oxide, nanowire, EDS mapping, short-circuit diffusion

## Abstract

Plenty of studies have been performed to probe the diverse properties of ZnO nanowires, but only a few have focused on the physical properties of a single nanowire since analyzing the growth mechanism along a single nanowire is difficult. In this study, a single ZnO nanowire was synthesized using a Ti-assisted chemical vapor deposition (CVD) method to avoid the appearance of catalytic contamination. Two-dimensional energy dispersive spectroscopy (EDS) mapping with a diffusion model was used to obtain the diffusion length and the activation energy ratio. The ratio value is close to 0.3, revealing that the growth of ZnO nanowires was attributed to the short-circuit diffusion.

## 1. Introduction

In the nanometer era, one-dimensional semiconductor nanomaterials with different morphologies have been fabricated by various methods and techniques. The properties of nanomaterials are usually quite distinct from these of bulk. Among the various semiconductors, zinc oxide (ZnO) has many charming properties that include a direct and wide band gap, large exaction energy, a large piezoelectric constant, strong ultraviolet emissions, stable structure, high penetrability, and good conductivity [1]. One-dimensional ZnO nanostructures have some potential applications such as nano-lasers, nano-detectors, and nano-sensors [2]. The ZnO nanowires can be fabricated by the vapor transport method [3,4], molecular beam epitaxy method [5], laser ablation method [6], simple thermal method [7,8], and so on. In recent years, the morphology, lengths, diameters, and growing directions of ZnO nanostructures could be roughly controlled by adjusting the parameters in the manufacturing process. Moreover, one-dimensional nanowire arrays [9], 3D network nanowires [10,11], and coaxial core–shell nanowires [12] have been synthesized using special methods with various catalysts and auxiliaries have been used in many of these methods. However, despite plenty of studies having been performed to probe the various properties of ZnO nanowires, only a few have specialized in investigating the optical properties of a single nanowire. Recent studies on ZnO nanowires have slightly shifted the focus to different aspects that include the growth mechanism, structural transformation, electron mobility, and phonon transmission. After understanding the disguised reaction mechanism, it could be possible to control and modify the electronic properties of nanowires. The mechanical characterization of single ZnO nanowire was reported by Argawal et al. [13,14], the size effect of Young’s modulus of ZnO nanowires was investigated experimentally, as well as computationally. Experimentally, ZnO nanowires with diameters ranging from 20.4 to 412.9 nm were tested under a uniaxial tensile load using a nanoscale materials testing system inside a transmission electron microscope, revealing the Young’s modulus of ZnO nanowires monotonically decreases from 160 to 140 GPa as the nanowires diameter increases from 20 to ∼80 nm. Therefore, to date, how to synthesize high-crystalline nanowires and how to establish an ordered investigation are very important scientific and technical issues. However, some challenges have to be overcome. Firstly, researchers have to develop various fabrication methods to investigate the properties of ZnO nanowires and to make large-area ZnO nanowire arrays for the purpose of potential applications. In most of these various synthesis methods, various catalysts or auxiliaries were utilized in these fabrication processes. Residual contaminations could be found on the surface or at the edge of the nanomaterials with the result that it is difficult to observe the essential properties of ZnO. Therefore, the synthesis of nanowires without a catalyst or using a one-step growth method would be of interest. These syntheses would be useful to directly characterize the physical properties. Moreover, the 3D network ZnO nanowires with high surface-to-volume ratio have attracted a great deal of attention, while how to control the dimensionality and size of ZnO nanowires has also become popular. Secondly, compared to the developed growth mechanisms for zero- and two-dimensional nano systems, the growth mechanism of nanowires deserves to be investigated in-depth. The growth mechanism of metal-oxide nanowires using catalytic methods is often attributed to the vapor-liquid-solid mechanism, but without using catalysts it is not clear. Some reporters have proposed that the metal-oxide nanowires grown below the melting temperatures are attributed to the short-circuit diffusion mechanism. Investigating the atomic diffusion in metal-oxide films is becoming an increasingly important issue. Lastly, plenty of studies have been performed to probe the diverse properties of ZnO nanowires, but only a few have focused on the physical properties of a single nanowire since analyzing the phonon confinement along a single nanowire is difficult. In addition to this, previous studies of size effects were usually investigated by fabricating various sized nanoparticles and examining these properties. Nevertheless, the multi-contributions of nanoparticle shapes, size distributions, thermal effects, surface effects, and strain have resulted in a complicated situation in which the experimental pieces of information could not be compared with one another [15]. The investigation of a single ZnO nanowire, therefore, provides a reasonable possibility for probing the size effect.

In this study, we report the syntheses of a single ZnO nanowire using a Ti-assisted chemical vapor deposition (CVD) method to avoid the appearance of catalytic contamination. The dimensionality and size of ZnO nanowires can be controlled through fabrication time. A two-dimensional energy dispersive spectrum is a conventional technique to examine the diffusion situation in nanostructures. A short-circuit diffusion model was presented.

## 2. Materials and Methods

One-dimensional ZnO nanostructures can be fabricated by various methods. Two typical methods are introduced as follows. The chemical vapor deposition (CVD) method with metal catalysts is a common method to fabricate semiconductor nanowires, such as TiO_2_, ZnO, GaN, and so on [4,16,17]. A typical example of ZnO nanowires synthesized by chemical vapor transport and a condensation system has been reported by Yang et al. [17]. During the process of nanowire growth, the zinc powder was heated to generate zinc vapor and then flowed to the substrate. The zinc vapor reacted with the gold solvent on the substrate at a lower temperature region to form alloy droplets. When the alloy droplet became supersaturated, crystalline ZnO nanowires were grown on the substrate surface. The oxygen atom source originated from the reaction between zinc and CO/CO_2_ vapor. The growth mechanism could be attributed to the VLS (vapor-liquid-solid) crystal growth mechanism which is widely used for explaining the growth mechanism of oxide nanowires.

Compared with the previous methods, the thermal evaporation method without using catalysts [18,19] is another simple method for the production of ZnO nanowires. The fabrication of ZnO nanowires under ambient air and collected products on the surface of the sample stage has been reported by Wang et al. [19]. No distinguishable suboxide (ZnO_1−*x*_) and impurity phase can be observed on the XRD patterns. In addition, 3D network ZnO structures can be fabricated by this method [20,21]. Chang et al. [20] fabricated ZnO nanowires using a chemical vapor deposition method at 700 °C under an argon gas flow in a quartz tube. The ZnO samples with different morphology were collected in various regions on the substrate. The authors proposed that the tuning pressure effect results in a different nucleation rate which further affects the morphology.

**Figure 1:**
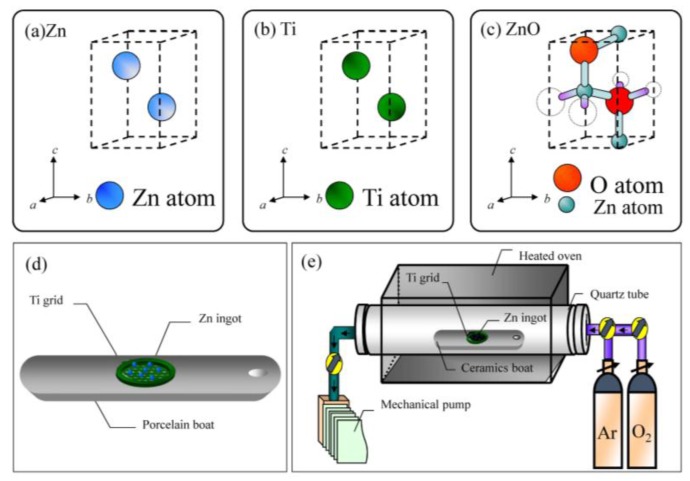
Schematic figures of unit cells of (**a**) zinc; (**b**) titanium and (**c**) ZnO; (**d**) the schematic figure for a porcelain boat, in which a high-purity zinc ingot (~0.2 g, 99.99%) on a cleaned Ti grid wafer was mounted on a cut silicon wafer; (**e**) the schematic figure for the chemical vapor deposition (CVD) instrument.

In this study, we used the CVD method to fabricate ZnO nanowires without an auxiliary template or any catalyst. Only a pure titanium grid (melting point: ~1941 K) was used as an auxiliary and substrate in this fabrication process. No other catalyst and auxiliary were used. The titanium is a good candidate for being a substrate due to both zinc and titanium having a hexagonal structure with a space group of *P6_3_/mmc*, while ZnO has a hexagonal structure with a space group of *P6_3_mc*, as shown in Figure 1a–c, respectively. As shown in the supplementary materials of Table S1 [22], the lattice constants are close with each other. In addition to this, no TiO*_x_* nanowires were grown on the surface of the Ti grid during the annealing process at a temperature range of 300–800 °C. Based on this, the titanium grid was selected as the substrate. Compared with other methods, this approach is a simple, convenient, and reliable method for preparing ZnO nanowires. The complete synthesis process is as follows: (1) a porcelain boat, a cut zinc ingot (0.2 g), and a pure silicon plate with a side length of 0.5 cm were cleaned with ethanol and then were washed in a low-energy ultrasonic cleaning bath for five minutes, respectively; (2) the zinc ingot was mounted on a pure titanium grid with a mesh number of 200; (3) the grid was put on the cleaned silicon plate and then all were placed on a porcelain boat as shown in Figure 1d; (4) the boat was placed in a quartz tube in the middle region of a heated oven as shown in Figure 1e; (5) the pressure of the quartz tube was reduced to less than 1 × 10^−2^ Torr by a mechanical pump; (6) the heating temperature in the quartz tube was set for various samples in a temperature range of 300–800 °C, respectively; (7) these temperatures were automatically adjusted by a current controller; (8) after the temperature was stabilized, a mixed gas of oxygen (20 *sccm*) and argon (80 *sccm*) was introduced into the tube and the pressure was kept at 760 Torr by a flux controller; (9) the boat was heated at a set temperature for two hours; (10) after heating, the samples were cooled to room temperature naturally after the heating; and (11) these as-grown samples were saved in a low-pressure container to avoid further oxidation. The zinc ingot on the grid melted in the heating process and then the liquid zinc uniformly covered the grid. The zinc atoms combined with oxygen atoms after which ZnO nanowires grew on the ZnO film. The optical images of various annealing temperatures samples were shown in the supplementary materials of Figure S1 [22]. As can be seen in Figure S1, the heated titanium grids were usually curly and fragile. These samples were characterized by various techniques and are discussed in further sections. In order to achieve the research purpose, we used several measuring instruments to characterize the properties of ZnO nanowires. The morphological appearance and elemental composition were characterized by field-emission scanning electron microscopy and an energy dispersion spectrometer, respectively. The atomic image and crystal structure were obtained by analytical transmission electron microscopy.

## 3. Results and Discussion

### 3.1. Morphological Analysis of ZnO Nanowire

**Figure 2:**
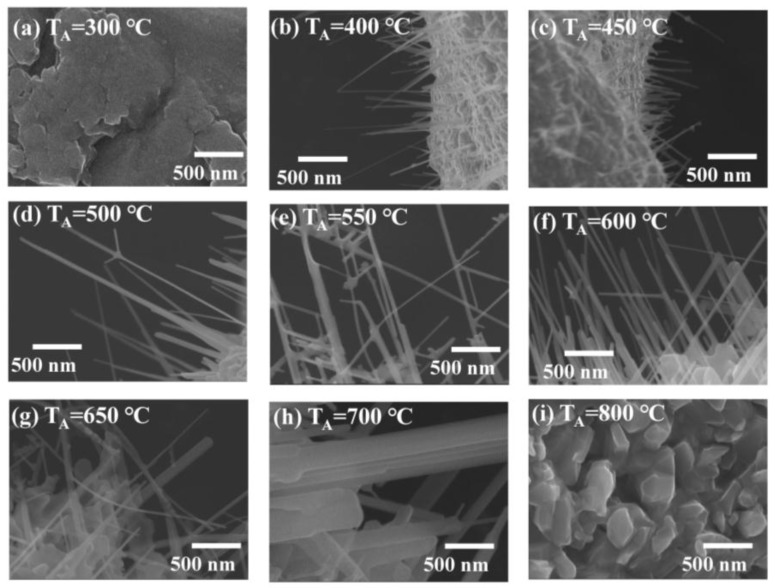
Scanning electron microscopy (SEM) images for a series of samples synthesized at (**a**) 300 °C; (**b**) 400 °C; (**c**) 450 °C; (**d**) 500 °C; (**e**) 550 °C; (**f**) 600 °C; (**g**) 650 °C; (**h**) 700 °C and (**i**) 800 °C, respectively.

**Figure 3:**
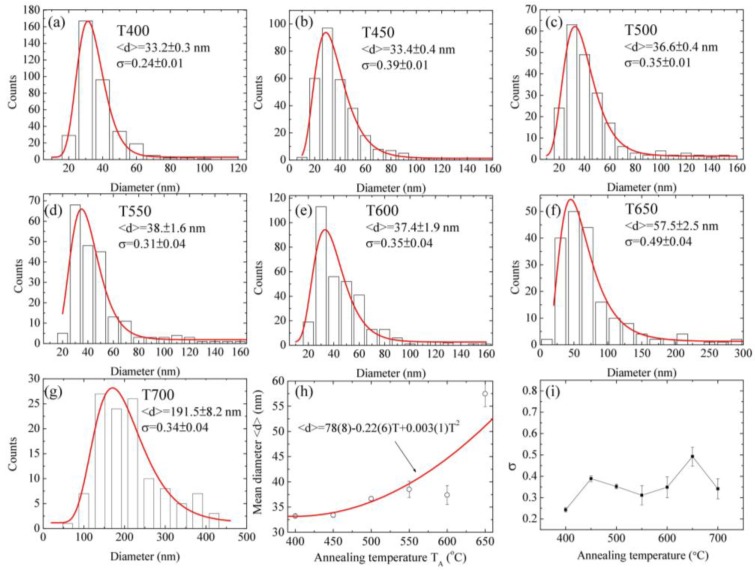
Size distributions for a series of samples synthesized at (**a**) 400 °C; (**b**) 450 °C; (**c**) 500 °C; (**d**) 550 °C; (**e**) 600 °C; (**f**) 650 °C and (**g**) 700 °C, respectively; and (**h**,**i**) show the mean diameter and the standard deviation versus annealing temperatures.

An electron microscope is a precision electro-optical instrument for observing the scattering between incident electrons and samples to investigate material morphology and fine structure. In this study, field-emission scanning electron microscopy (FE-SEM, JEOL, JSM 6500F, Peabody, MA, USA) was used to characterize the morphology of ZnO nanowires. In this study, the elemental component and elemental mapping of ZnO nanowires was observed by energy-dispersive X-ray spectroscopy (Inca X-sight model 7557 Oxford Instrument, Abingdon, Oxfordshire, UK) that was equipped with the above-mentioned scanning electron microscope. The energy resolution is less than 133 eV. The detectable range of elements is from boron to uranium. The maximum detectable energy of X-rays is related to the incident electron beam energy. Following the SEM process, 15 keV is the limit. The sample preparation and experimental process are similar to the steps for SEM measurement. Figure 2a–i displays these SEM images with a magnification of 50,000 for various samples synthesized at annealing temperatures of 300–800 °C, respectively. In the further sections, the sample synthesized at 300 °C is labeled as T300, and other samples are tagged in the same way. It can be seen that, the ZnO nanowires only can be seen on the samples of T400–T700. The one-dimensional ZnO nanowires were observed on the surface of T400 and T450 and 3D network ZnO nanowires were obtained in a temperature range of 500–700 °C. In the one-dimensional ZnO samples as shown in Figure 2b,c, the ZnO nanowires were individually separated and straight. The diameters and lengths of the ZnO nanowires were in the ranges of a few tens of nanometers and several μm, respectively. In the three-dimensional (3D) ZnO samples, as shown in Figure 2d–h, the branching ZnO nanowires were found on the grid surface. The branches grew randomly on the trunk and were not perpendicular to the truck. With the annealing temperature increasing, the branch numbers and structural complexity clearly increased. As the annealing temperatures were lower than 400 °C or higher than 700 °C, no nanowire can be seen on the grid surface. With the high temperature range shown in Figure 2i, the surfaces become very rough as a result of grain growth. The grain size is in a range from 100 nanometers to 5 μm. The diameter distribution of ZnO nanowires could be described using a log-normal function. We measured the diameters for various samples and fitted the curves using a log-normal function, respectively. The log-normal function is defined as follows: $f(d)=\frac{1}{{\left(2\pi \right)}^{0.5}d\sigma }\exp[-\frac{{\left(\ln d-\ln<d>\right)}^{2}}{2\sigma^{2}}]$, where *d* is the diameter, <*d*> is the mean value and σ is the standard deviation. The experimental data and fitting curves were plotted by black hollow bars and red solid lines in Figure 3a–g, respectively. The corresponding fitting parameters are shown in Table 1. For the sample of T400, as an example, the mean value is 33.2 ± 0.3 nm and the standard deviation is 0.24 ± 0.01. The low standard deviation (<0.5) means that the nanowire size is confined in a small range. The regression constant R^2^ of 0.99 indicates the fitting curve is very close to the size distribution. From the overall results, the mean diameter of ZnO nanowires reveals an obvious dependence on annealing temperature as shown in Figure 3h; the mean diameter increases with increasing annealing temperatures. In the temperature range of 400–650 °C, the temperature dependence of growth mean diameter of ZnO nanowires can be described by a parabolic law (solid curve). The standard deviations as shown in Figure 3i are less than 0.5, revealing that the distribution of mean diameters is uniform.

### 3.2. Crystal Structure Analysis of ZnO Nanowires

**Figure 4:**
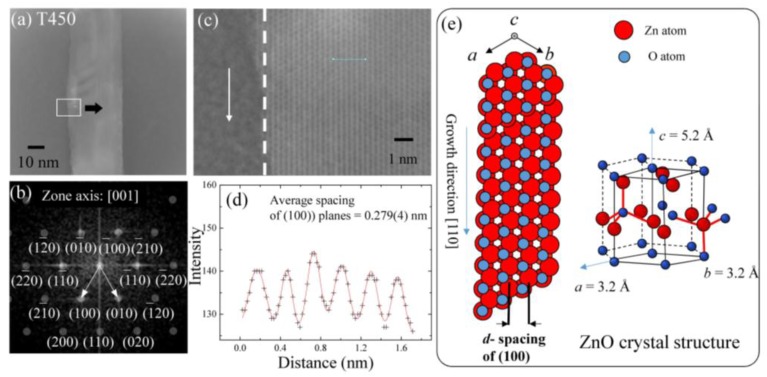
(**a**) Transmission electron microscopy (TEM) image; (**b**) corresponding selected-area electron pattern; (**c**) high-resolution image of the selection region (marked in (**a**)) of ZnO nanowires for T450; (**d**) height-position intensity along the line taken from high resolution (HR)-TEM (marked in (**b**)); and (**e**) schematic figure of the crystal structure for a single ZnO nanowire.

Transmission electron microscopy (TEM) is an important microscopic technique for cancer research, material science, and nanotechnology. In the present study, the high-resolution (HR) images and the selected area electron diffraction (SAED) patterns were obtained by an analytical transmission electron microscope (JEOL 3010, Peabody, MA, USA) and a field emission transmission electron microscope (JEOL JEM-2100, Peabody, MA, USA). Figure 4a shows an example of the TEM result of a straight ZnO nanowire of the T450 sample. The diameter of the ZnO nanowire is about 32 nm. The single crystalline nature is revealed. The diffraction pattern along the (001) direction shown in Figure 4b can be indexed as hexagonal ZnO in structure with a space group of *P6_3_mc*. A high-magnification enlargement of a selected area of the high-resolution TEM image is shown in Figure 4c. It can be seen that the normal direction of planes is not parallel to the growth direction of the ZnO nanowire. A gray-level analysis was used to extract the scattering intensity which can be fit using a multi-Gaussian function to obtain the average plane spacing. Figure 4d shows the fitting result, in which the asterisk and the solid line show the experimental data and fitting curve, respectively. The fitted distance is 0.279 (4) nm, corresponding to the *d*-spacing of (100) planes of ZnO hexagonal structure. The lattice parameter of the hexagonal structure can be calculated using the relationship: $\frac{1}{d_{hkl}{}^{2}}=\frac{4}{3}\frac{h^{2}+hk+k^{2}}{a^{2}}+\frac{l^{2}}{c^{2}}$, where *d*_hkl_ is a spacing between two planes of (*hkl*), *a* and *c* are lattice parameters, and *h*, *k*, and *l* are the Miller indices. The result shows the lattice parameter *a* of 0.32(3) nm. Since the *c*-axis of the hexagonal structure is perpendicular to the scattering plane, we could not measure the lattice constant *c.* Based on this analysis, a schematic crystal structure of a single ZnO nanowire is shown in Figure 4e. The growth direction of ZnO nanowires is along the (110) direction. As the annealing temperatures was higher than 500 °C, dendritic ZnO nanowires could be easily seen in the TEM images of Figure S2a (see supplementary materials of Figure S2) [22], showing the morphology of a typical dendritic ZnO nanowire. The Bragg spots as shown in Figure 2b correspond to the zone axis (001) reflection of the ZnO wurtzite structure.

The detailed structure of the ZnO nanowire was investigated using high-resolution images. Two high-magnification enlargements of selected regions, marked in Figure S2a, are shown in Figure S2c,d, respectively. It can be seen that there is an obvious atomic arrangement of hexagonal symmetry and the atomic spacing can be obtained by the above gray-level analysis. The values obtained from the fittings, as shown in Figure S2e,f were, respectively, 0.33 (3) and 0.33 (1) nm, corresponding to the lattice constant *a* of the ZnO wurtzite structure. This result of lattice parameters is consistent with that obtained by SAED observations. The growth directions of these branches are indicated by the (110) direction. It can be explained that the two nanowires were inclined towards each other by 60 degrees. In our experience, the branching nanowires appear randomly on the surface of dendritic ZnO nanowires and the angle between the branches and trunks is closed to multiples of 60 degrees and with no other angle able to be found [23,24,25]. Details of the corresponding lattice parameters for various samples are summarized into Table 2. As can be seen in Table 2, the lattice parameters of various samples are slightly smaller than that of bulk [26], assumed that the strain effect is responsible for the lattice contraction [27].

The ZnO nanowires have a growth direction of (110), which is in contrast to the common (001) growth direction [28,29,30,31]. Only a few papers [32,33] have reported that the ZnO nanowires grew along the (110) direction. Similar analysis method of the structure and lattice parameters for a single nanowire has been reported by Barriga et al. [34]. Four different cylindrical nanowire systems (Ni, Co and Co_58_Ni_42_/Co_83_Ni_17_ nanowires), grown by standard electrodeposition techniques in the nanometer size channels of porous alumina templates, were investigated using TEM and SAED. In their comprehensive analysis, these results can be explained by considering the characteristics of the measurement technique and the confined template-assisted growth, which force the atoms to be accommodated in a cylindrical volume with nanoscale dimensions. The growth mechanism of ZnO nanowires could be attributed to the short-circuit diffusion [33,35], the high zinc vapor pressure [36] and the diffusion-limited supersaturated environment [23,25]. Rackauskas et al. [33] assumed that the growth of ZnO nanowires is related to the diffusion through grain boundaries in the ZnO layer and the crystal defects in ZnO nanowires. Fan’s group [36] proposed that the growths of the trunk and branches go through a self-catalytic liquid-solid and vapor-solid process, respectively. Zinc atoms were heated to form a vapor at 600 °C and then nucleated on the nanowire surface to form branches. Complementarily, Park et al. [25] assumed that the supersaturated reactant vapors play an important role in forming the dendritic side branches. In this point of view, a catalyst is not necessary in the formation of branch growth and the growth process of ZnO nanowires should be dependent on a surface diffusion, with respect to the annealing temperatures and growth times. The formation of ZnO nanowires was carried out by a two-dimensional EDS mapping investigation.

### 3.3. Two-Dimensional EDS Mapping

**Figure 5:**
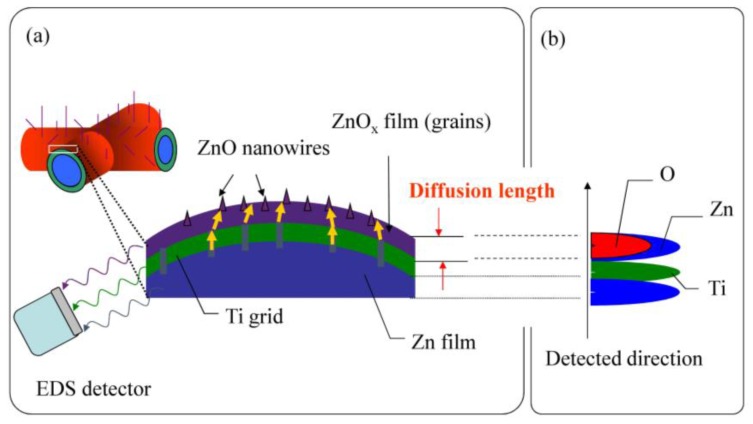
(**a**) Schematic diagram of EDS mapping; and (**b**) hypothetical results of the corresponding EDS spectra along the line scan.

To observe element and component distribution is important for investigating the diffusion in nanomaterials. There are numerous experimental methods, such as secondary ion mass spectrometry, electron microprobe analysis, auger electron spectroscopy, nuclear reaction analysis, nuclear magnetic relaxation, confocal Raman spectroscopy, transmission electron microscopy, and energy dispersive spectroscopy, for studying diffusion in solids, in which the energy dispersive spectrometer is a convenient and useful tool to analyze spatial element distributions, especially EDS mapping images that can offer direct evidence of element distributions. Figure 5a shows the schematic illustration of EDS mapping of the cross-sections of ZnO samples. The broken samples were fixed on cleaned silicon plates by carbon tape and then were mounted on a special sample holder for taking cross section images. An EDS detector scans an edge portion near the sample surface and then depicts a corresponding element mapping of a cross-section of the ZnO samples. Along the normal direction of the sample surface, we assumed that the bottom part is titanium substrate, followed by pure Zn film, ZnO_x_ film, and ZnO nanowires as shown in Figure 5b.

**Figure 6:**
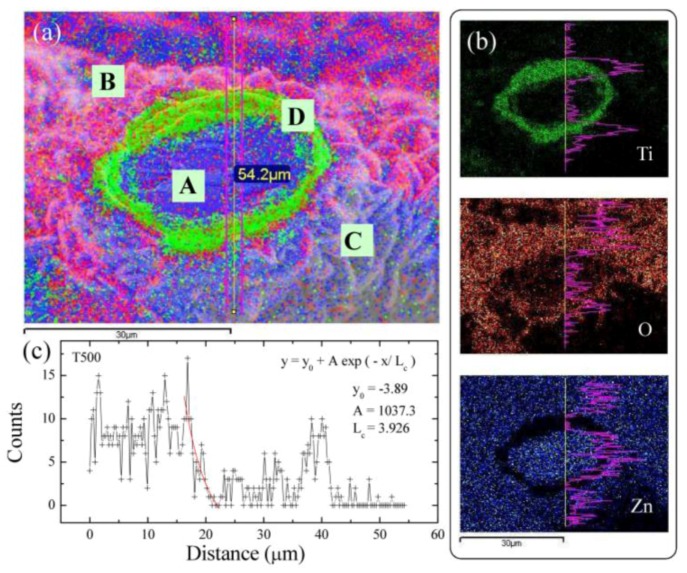
(**a**) A selected area EDS mapping of T500; (**b**) corresponding EDS mappings for a single element; and (**c**) the counts of oxygen versus the positions.

We assumed that the zinc atoms were diffused from the bottom of the ZnO films to the surface, and then formed the ZnO nanowires. In this view, the diffusion length is related to the thickness of layers on the grid surface. We can estimate the ZnO thickness to understand the growth mechanism through EDS mapping technique. Figure 6a shows an example of the EDS mapping result for T500 sample, in which the corresponding SEM image is used as a background. The size of the scanning area is about 60 × 54.2 μm, covering a cross-sectional area of a tube of titanium grid. The number of scanning times is 5 in order to improve the measurement accuracy. The elements of zinc (blue), oxygen (red), and titanium (green) were, respectively, indicated using the lock-in energy of Zn–*L*_α_ (0.8–1.2 keV), O–*K*_α_ (0.4–0.6 keV), and Ti–*K*_α_ (4.3–4.7 keV). As shown in the Figure 6a, the center of the tube (point A) shows the blue color, revealing the existence of the zinc component.

**Figure 7:**
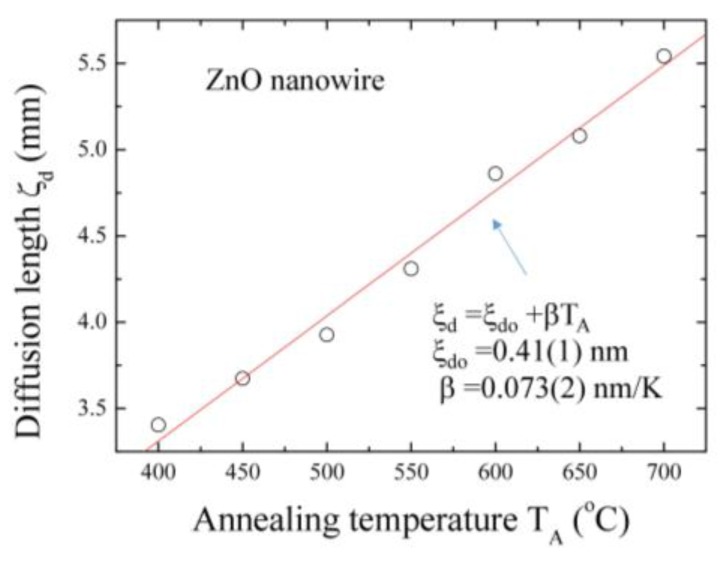
Diffusion length is dependent on the annealing temperature, revealing a growth rate of 0.0073 μm/°C^−1^.

We assume that, due to thermal heating and grid expansion, zinc flows into the hollow tube from the surface through crevices and fills the tube. On the upper surface of the grid (point B), the red dots and blue dots are distributed uniformly, revealing that the grid has been covered in a thin zinc oxide layer. On the other side of the grid surface (point C), the fewer oxygen signals were attributed to the smaller contact area with oxygen during the annealing process. At the edge of the grid surface (point D), a green ring showed clearly the position of the grid section. Incidentally, the number of signal points in the upper part of the Figure 6a is more than that in the lower part due to the detection angle and the focal length. The three element distribution mappings for Ti, O and Zn are, respectively, shown in Figure 6b, in which the purple curves show the intensities of each element along the vertical line (yellow), respectively. Line profile EDS analysis clearly shows the presence of O in the sample. It can be seen that the intensity of oxygen signals deceases suddenly near the wall of the Ti and the decreasing curve shown in Figure 6c can be used to obtain the thickness of ZnO layers. The curve can be obtained by an exponential function [37]. The line width of the distribution can be used to define the mean diffusion length <ξ_d_>. The obtained diffusion length ξ_d_ is near 3.40 μm for T500 sample. A series of EDS mapping were examined and obtained on various samples. The corresponding diffusion lengths of the ZnO layer versus the annealing temperatures are shown in Figure 7. As seen in Figure 7, the estimated values of ξ_d_ versus the annealing temperature T_A_ are plotted, revealing an increase with the increase in the diffusion length ξ_d_. The red solid curve indicates the fit of the data to the theoretical curve for an exponential decay function, namely ξ_d_ = ξ_do_ + βT_A_, where ξ_do_ = 0.41 (1) nm and β = 0.073 (2) nm/K represents the initial constant and the fitted parameters, respectively.

In general, the short-circuit diffusion plays an important role below 0.5 times the melting temperature [38]. The various samples in this study were fabricated below 0.5 times the melting temperature of ZnO (1975 °C) [26], in which recrystallization and grain growth proceed slowly and polycrystallinity provides effective short-circuit diffusion paths [35,39]. The short-circuit mechanism was used to describe the formation of one-dimensional nanostructures [40]. For example, Lu et al. [41] fabricatedα–Fe_3_O_4_ nanowires by oxidizing iron in pure oxygen between 400 and 600 °C. The oxide layer can be controlled by varying the oxidation temperatures to form grains. They pointed out that the iron ions diffuse from the Fe_2_O_3_/Fe_3_O_4_ interface to the free surface via grain boundary diffusion. Subsequently, the Fe ions diffuse from the grain boundary to the nanowire root via surface diffusion to form Fe_3_O_4_ nanowires on the top of Fe_3_O_4_ grains.

**Figure 8:**
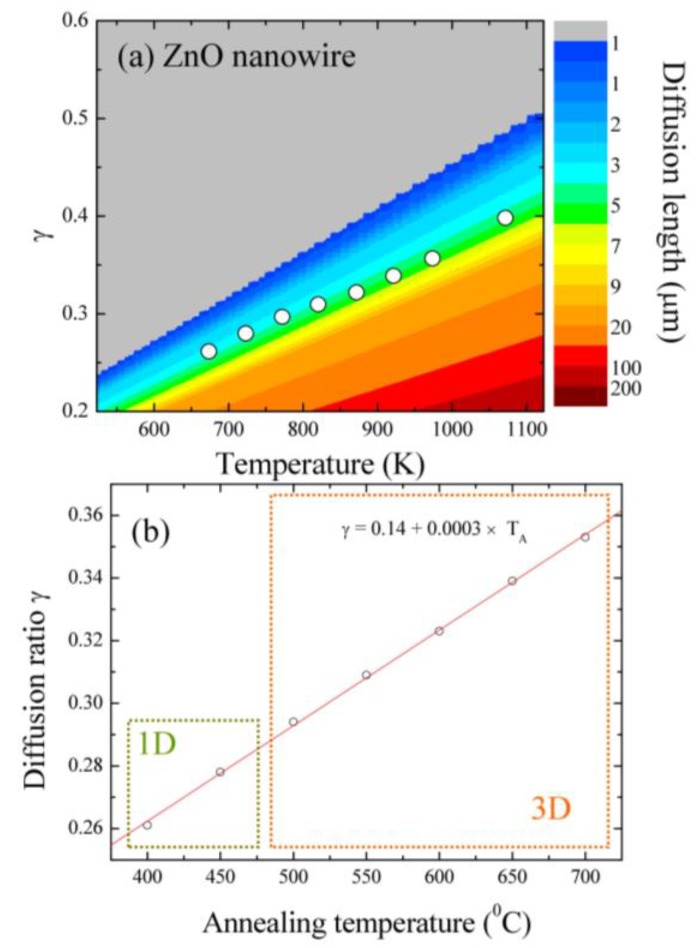
(**a**) Plots of the diffusion length and diffusion ratio γ versus the annealing temperatures; and (**b**) the diffusion ratio γ versus the annealing temperatures.

Xu et al. [42] fabricated CuO nanowires on Cu foils in wet air at a temperature range of 400–700 °C, with diameters between 50 to 400 nm and lengths between 1 and 15 micrometers. The authors assumed that a high density of sub-boundaries in the surface layer enhances the formation of nanowires. They emphasized that the short-circuit diffusion dominates in the middle temperatures, while the lattice diffusion would be important at high temperatures. Yuan et al. [43] proposed a method to enhance the nanowire growth density and length by increasing the surface roughness. It was found that the increased surface roughness (smaller grains) results in more short diffusion paths and surface sites that contribute to the diffusive transport of copper atoms along grain boundaries and the nucleation of CuO in order to improve the nanowire density and length. We purposed that the growth mechanism of ZnO nanowires was attributed to the short-circuit diffusion. A diffusion model [44] was used to interpret the diffusion length, where a parameter of γ is taken as the percentage of the lattice activation energy of zinc ions to clarify the contribution from short-circuit- or lattice-diffusion. The values γ of 1/3 and 1 indicate that the diffusion prefers short-circuit- and lattice-diffusion, respectively [35]. The equation of diffusion length is shown as follows: $\Delta L=\sqrt{D_{L}\cdot \tau }={\left[\frac{\beta }{2}\alpha^{2}v_{D}\exp(-\frac{\gamma Q}{RT})\cdot \tau \right]}^{1/2}$, where Δ*L* is the diffusion length, *D_L_* is the diffusion coefficient, *τ* is the growth time (approximately 7200 s), *β* is the number of atoms jumping along (110), α (= 0.162 nm) is the *d*-spacing of (110) related to the diffusion direction, *v*_D_ (= 1.73 × 10^11^ s^−1^) is the vibrational frequency, *Q* (= 318 kJ/mol) is the activation energy of ZnO [45], *R* (= 1.987 cal·mol·K^−1^) is the gas constant, and *T* is the growth temperature. The vibrational frequency can be calculated by the equation: $v_{D}=\frac{1}{2^{1/2}}\left(\frac{Q}{m\alpha^{2}}\right)$, where *m* (= 65.4 g/mol) is the zinc molar weight. Figure 8a shows the diffusion ratio γ versus annealing temperatures. The colors indicate the different diffusion lengths as shown on the left, in which the gray color denotes a diffusion length of less than 1 μm and the red color represents that of more than 200 μm. The obtained diffusion lengths are marked in the figure by white solid circles. It can be seen that the diffusion lengths corresponding to the growth temperatures of ZnO nanowires are located in the region of 0.26–0.35 times the activation energy of ZnO lattice diffusion, revealing that short-circuit diffusion dominates the diffusion process. Moreover, the value of activation energy at T400 sample is ~83 kJ/mol, close to the migration energies of zinc interstitials (77 kJ/mol) and vacancies (88 kJ/mol) [46]. Figure 8b and Table 3 show the diffusion ratio γ versus the annealing temperatures, in which the experimental data can be described by a linear function.

**Figure 9:**
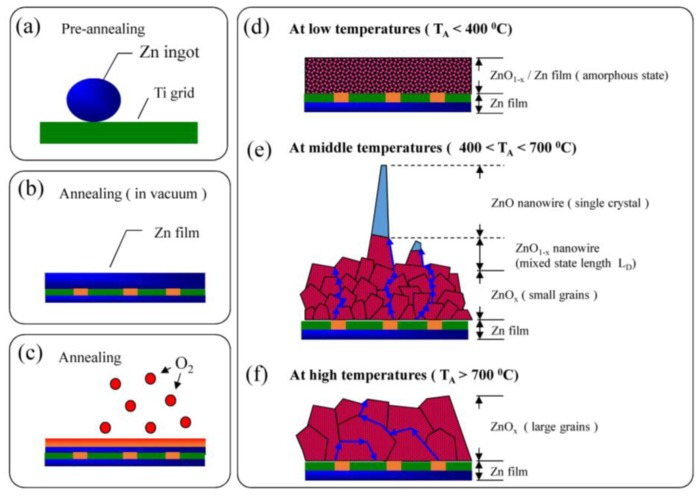
A schematic plot of growth process of ZnO structures: (**a**) sample preparation before annealing; (**b**) the formation of zinc film; (**c**) the oxidation on the film surface and the formation of various ZnO structures at (**d**) T_A_ < 400 °C, (**e**) 400 < T_A_ < 700 °C, and (**f**) T_A_ > 700 °C.

In the high-temperature range, the diffusion ratio is high, meaning that the lattice diffusion plays an important role in the transport process. The ZnO nanowires were found in a γ range of 0.261–0.353 where these γ values are close to 0.3, revealing that the short-circuit diffusion mechanism dominates the diffusion. The dimensionality of ZnO nanowires can be controlled by adjusting the annealing temperatures and the diffusion ratio. Figure 9 shows the schematic illustration of the growth process. In the first step, the zinc ingot melted to form a zinc film in a reduced oxygen environment, as shown in Figure 9a. Then, the zinc film reacted with the introduced oxygen gas to construct a thin ZnO film on the top of the zinc film, as shown in Figure 9b. The thickness of the ZnO layer could be obtained by probing the oxygen distribution in EDS mapping, as shown in Figure 9c. Finally, zinc atoms could diffuse through the ZnO film to form various structures on the surface at temperature regions of T_A_ < 400 °C (Figure 9d), 400 < T_A_ < 700 °C (Figure 9e), and T_A_ > 700 °C (Figure 9f), respectively, in which the ZnO nanowires were found at middle temperatures and ZnO film was obtained both at high and low temperatures. The contribution of oxygen migration is not considered due to the large atomic size and high migration energies [32]. According to the previous report [33], the migration energies of oxygen interstitial and vacancy are 118 and 124 kJ/mol, respectively. ZnO nanowires cannot grow at high temperatures, which could be explained by the grain sizes and thickness of ZnO*_x_*. It is well known that the contribution from boundary diffusion decreases with increasing nanocrystal size [47,48]. Brass and Chanfreau [48] proposed that a large density of grain boundaries would provide fast atomic diffusion paths along the boundaries. Based on this, atoms that diffuse through small grains to the surface would be faster than those in large grains. The grain size increases from tens to hundreds of nanometers with increasing annealing temperatures. Besides this, the thickness of ZnO*_x_* films also increased with annealing temperatures that were obtained in EDS mapping. Both the large grains and thick films fabricated at high temperatures would prevent zinc atomic diffusion along grain boundaries from the zinc film to the surface to form ZnO nanowires, in which the lattice diffusion would be the dominant transport mechanism.

## 4. Conclusions

The growth mechanism and the phonon confinement effect in 1D and 3D network ZnO nanowires are investigated. The ZnO nanowires were fabricated by a Ti-assisted chemical vapor deposition method without any catalyst in a temperature range of 400–700 °C. The mean diameter ranging from 33.2 to 191.5 nm increased with the increasing temperature. The dimensionality can be controlled by adjusting the annealing temperatures. Below 500 °C, only one-dimensional ZnO nanowires can be found on the sample surface, and, above it, three-dimensional ZnO nanowires were able to be grown. ZnO nanowires have a hexagonal structure with a space group of *P6_3_mc* and the (110) growth direction. The formation of ZnO nanowires was attributed to the short-circuit diffusion. Energy dispersive X-ray spectroscopic mapping technique was used to depict the diffusion of zinc atoms through ZnO*_x_* film from the zinc base to the film surface. A diffusion model was utilized to calculate the activation energy of the diffusion for various samples. The result shows that the activation energy is 0.26–0.35 times the activation energy of ZnO lattice diffusion, revealing that the growth of ZnO nanowires was related to the diffusion goes through the grain boundaries or sub-boundaries and then forms ZnO nanowires.

## Figures and Tables

**Table 1:** Summary of the fitting parameters obtained from the log normal function.

T_A_ (°C)	Sample	<d> (nm)	δ (nm)	Area	R^2^
400	T400	33.2 ± 0.3	0.24 ± 0.01	3219 ± 113	0.99
450	T450	33.4 ± 0.4	0.39 ± 0.01	2789 ± 78	0.99
500	T500	36.6 ± 0.4	0.35 ± 0.01	1834 ± 204	0.99
550	T550	38.5 ± 1.6	0.31 ± 0.04	1834 ± 204	0.92
600	T600	37.4 ± 1.9	0.35 ± 0.04	2817 ± 331	0.88
650	T650	57.5 ± 2.5	0.49 ± 0.04	3348 ± 441	0.97
700	T700	191.5 ± 8.2	0.34 ± 0.04	4177 ± 442	0.93

**Table 2:** A summary of lattice parameters for various samples.

Sample	Trunk		Branch	
	Diameter (nm)	Lattice Constant *a* (nm)	Diameter (nm)	Lattice Constant *a* (nm)
T400	13.0	0.32 (2)		
T450	32.1	0.32 (3)		
T500	54.1	0.33 (1)	19.1	0.33 (3)
T550	37.1	0.32 (3)	25.8	0.32 (3)
T600	65.2	0.32 (2)	56.4	0.32 (6)
T650	83.3	0.32 (3)	33.3	0.32 (3)

**Table 3:** Diffusion lengths along with simulated results for a diffusion theory. The value γ is the ratio of the activation energy.

Dimensionality	Sample	Diffusion Length (μm)	Diffusion Ratio γ
1-D	T400	3.404	0.261
1-D	T450	3.674	0.278
3-D	T500	3.927	0.294
3-D	T550	4.310	0.309
3-D	T600	4.861	0.323
3-D	T650	5.080	0.339
3-D	T700	5.542	0.353
Large grains	T800	4.462	0.402

## Supplementary Materials

The following are available online at http://www.mdpi.com/2079-4991/7/7/188/s1, Table S1. A list of space groups and lattice parameters of Zn, Ti and ZnO; Figure S1. Optical images for various annealing temperaure of samples (a) an unheated Ti grid and a series of samples synthesized at (b) 300 °C; (c) 350 °C; (d) 400 °C; (e) 450 °C; (f) 500 °C; (g) 550 °C; (h) 600 °C; (i) 650 °C; (j) 700 °C; (k) 750 °C; and (l) 800 °C, respectively; Figure S2. (a) TEM image; (b) corresponding selected-area electron pattern; (c,d) high-resolution images of selected regions (marked in (a)) of ZnO nanowires for T500; (e) and (f) show the height-position intensity along the lines taken from HR-TEM marked in (c) and (d), respectively.
