# Supplementary material for: Growth Mechanism Studies of ZnO Nanowires: Experimental Observations and Short-Circuit Diffusion Analysis
Source: Nanomaterials (Basel). 2017 Jul 21;7(7):188. doi: 10.3390/nano7070188 (PMC5535254; doi:10.3390/nano7070188)
Supplement: Supplementary file 1 [file nanomaterials-07-00188-s001.pdf]

nanomaterials

Supplymentary information

Growth Mechanism Studies of Multi-Dimensional ZnO Nanowires: Experimental Observations and Theoretical Simulations

Po –Hsun Shih and Sheng Yun Wu*

Department of Physics, National Dong Hwa University, Hualien 97401, Taiwan

Table S1. A list of space groups and lattice parameters of Zn, Ti and ZnO.

Hexagonal Structure				
	Sspace group	a(nm)	c(nm)	Angles
Zn	$P6_3/mmc$	0.267	0.494	α=β=90°, γ =120°
Ti	$P6_3/mmc$	0.295	0.469	α=β=90°, γ =120°
ZnO	$P6_3mc$	0.324	0.520	α=β=90°, γ =120°

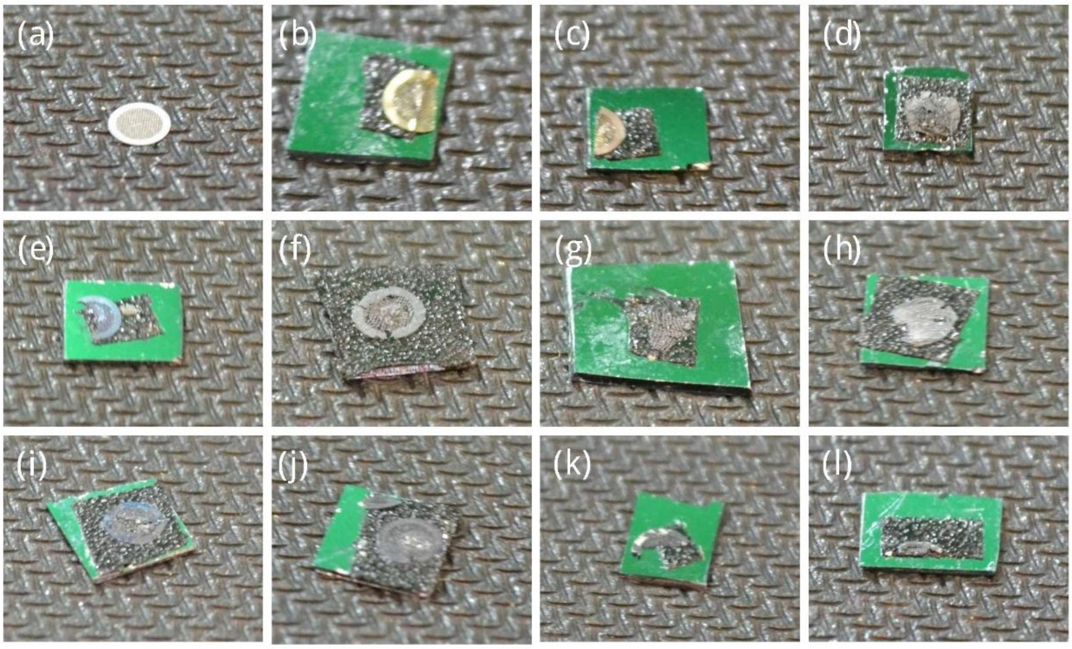

Figure S1. Optical images for various annealing temperaure of samples (**a**) an unheated Ti grid and a series of samples synthesized at (**b**) 300 °C; (**c**) 350 °C; (**d**) 400 °C; (**e**) 450 °C; (**f**) 500 °C; (**g**) 550 °C; (**h**) 600 °C; (**i**) 650 °C; (**j**) 700 °C; (**k**) 750 °C; and (**l**) 800 °C, respectively.

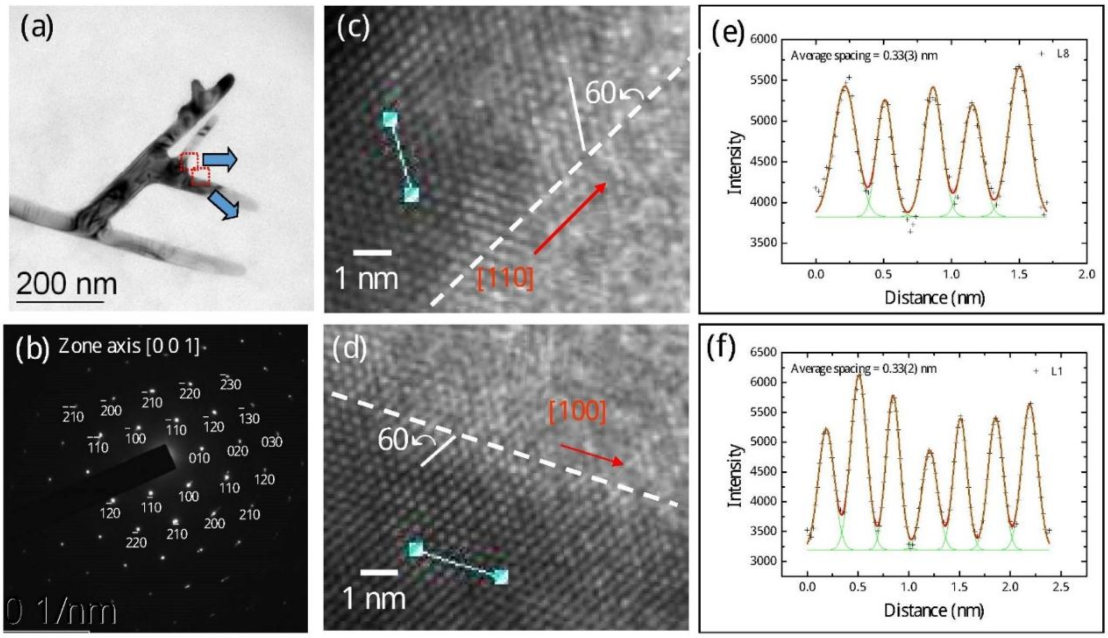

Figure S2. (**a**) TEM image; (**b**) corresponding selected-area electron pattern; (**c–d**) high-resolution images of selected regions (marked in (a)) of ZnO nanowires for T500; (**e**) and (**f**) show the height-position intensity along the lines taken from HR-TEM marked in (c) and (d), respectively.

© 2017 by the authors. Submitted for possible open access publication under the terms and conditions of the Creative Commons Attribution (CC BY) license (http://creativecommons.org/licenses/by/4.0/).
